# Methoxyquinolone–Benzothiazole Hybrids as New Aggregation-Induced Emission Luminogens and Efficient Fluorescent Chemosensors for Cyanide Ions

**DOI:** 10.3390/ijms252312896

**Published:** 2024-11-30

**Authors:** Mario Mutis-Ayala, Jorge Trilleras, Richard D’Vries, Mario A. Macías, Alberto Insuasty, Rodrigo Abonia, Jairo Quiroga, Luis A. Illicachi, Edgar Márquez, Daniel Insuasty

**Affiliations:** 1 Grupo de Investigación en Compuestos Heterocíclicos, Universidad del Atlántico, Puerto Colombia 081007, Colombia; mmutisayala@mail.uniatlantico.edu.co (M.M.-A.); jorgetrilleras@mail.uniatlantico.edu.co (J.T.); 2 Grupo de Investigación en Química de Productos Naturales, Departamento de Química, Facultad de Ciencias Naturales, Exactas y de la Educación, Universidad del Cauca, Calle 5 # 4–70, Popayán 190003, Colombia; richard.dvries@unicauca.edu.co; 3 Cristalografía y Química de Materiales, CrisQuimMat, Facultad de Ciencias, Departamento de Química, Universidad de los Andes, Cra. 1 #18a-12, Bogotá 111711, Colombia; ma.maciasl@uniandes.edu.co; 4 Grupo de Investigación de Compuestos Heterocíclicos, Departamento de Química, Universidad del Valle, Calle 13 # 100–00, Cali 760032, Colombia; alberto.insuasty@correounivalle.edu.co (A.I.); rodrigo.abonia@correounivalle.edu.co (R.A.); jairo.quiroga@correounivalle.edu.co (J.Q.); 5 Grupo de Investigación en Química y Biotecnología, Facultad de Ciencias Básicas, Universidad Santiago de Cali, Calle 5 # 62-00, Cali 760035, Colombia; luis.illicachi00@usc.edu.co; 6 Departamento de Química y Biología, División de Ciencias Básicas, Universidad del Norte, Km 5 vía Puerto Colombia, Barranquilla 081007, Colombia; ebrazon@uninorte.edu.co

**Keywords:** quinolone, benzothiazole, aggregation-induced emission, cyanide, chemosensors

## Abstract

This work describes the synthesis and characterization of new quinolone–benzothiazole hybrids, the study of their aggregation-induced emission (AIE) properties, and the use of these systems as efficient fluorescent probes for cyanide ions. These conjugated derivatives are linked through a double bond favoring electronic communication, and together with their planar geometry, can strongly aggregate under solvophobic environments, leading to aggregation and exhibiting significant AIE behavior. The double bond between electroactive units is prone to nucleophilic addition reactions by cyanide ions, selectively, conducive to turning off the fluorescence properties, making this hybrid system an efficient probe for cyanide ions. These studies were theoretically explained using DFT and TD-DFT calculations.

## 1. Introduction

Quinolone derivatives belong to a special class of heterocyclic compounds exhibiting remarkable pharmacological activities, especially as anticancer, antibacterial, antifungal, antioxidant, and anti-inflammatory agents [1,2,3,4,5,6]. The synthetic versatility of quinolones from a chemical point of view has led to access to a broad variety of derivatives based on this scaffold with several applications in different fields. Among those, quinolone derivatives have shown interesting fluorescent properties that have been used for bio-imaging, molecular probing, and chemosensory purposes with promising results [7,8,9,10].

Another interesting class of heterocyclic compounds, benzothiazole derivatives, have recently attracted considerable attention in the scientific community due to their application as promising pharmacophore platforms, exhibiting striking activities as potent anticancer, antibiotic, antimicrobial, and antituberculosis agents [11,12,13]. Benzothiazole derivatives have also been used as sensors, leading to the detection of certain biological species or ions [14,15,16,17,18]. Photophysical mechanisms like excited-state intramolecular photon transfer (ESIPT), photoinduced electron transfer (PET), intramolecular charge transfer (ICT), and aggregation-induced emission (AIE), along with benzothiazole-based fluorescent probes, can selectively interact with a broad range of analytes leading to a change in their emission properties that enables the efficient detection of the analyte. Moreover, benzothiazole moiety has also been used for the design of fluorescent probes for bioimaging applications [19,20].

Among all the previously mentioned photophysical mechanisms, AIE has attracted a huge interest as this property emerges from aggregated materials [21,22,23]. The AIE mechanism was first described by Tang et al. and is related to a process called restricted intramolecular motions (RIM), which can be favored by self-aggregation, solid state, solvophobic interactions, and excimer formations [23,24]. AIE has found applications in bioimaging and theragnostics due to the notable photostability of such aggregates exhibiting high fluorescent quantum yields. Moreover, AIE phenomena have also been used for sensing, nanomedicine, and organic solar cells [25,26,27,28,29,30,31].

Now, sensing certain anions from fluorescent probes is highly desirable for a broad range of applications [32,33,34]. It is well known that anions play key roles in chemical and biological processes and have been well-studied in several areas such as life sciences, chemistry, medicine, catalysis, and environmental sciences [1,35,36,37]. Some of them come mainly from industrial sources, and sometimes interrupt the proper balance in nature and thus generate undesired consequences for ecosystems. In this sense, the recognition of specific anions, mainly those considered dangerous, has become an essential issue among researchers. In particular, the recognition of cyanide (CN^−^), is quite important since an undesired discharge of this anion in nature can result not only in significant environmental risk, but also it is considered very harmful to humans even at a dosage of only 0.5 mg per kilogram of body weight [38,39]. Thus, being able to detect cyanide anions through efficient sensors is highly desirable. In this regard, florescent chemosensors are becoming more attractive due to their remarkable selectivity, versatility, low cost, less complex sample preparation, and quick reaction times, which enable real-time detection. All these features make them good candidates as an alternative to other analytical techniques used for detecting cyanide ions, such as electrochemistry, polarography, and gas chromatography [40,41,42,43].

In this work, we describe the design and synthesis of quinolone–benzothiazole hybrids as notable AIE luminogens, and we study their use as efficient cyanide ion chemosensors. Both electroactive units are linked through a double bond favoring electronic communication. The planar geometry of these molecules together in solvophobic environments leads to their strong aggregation, exhibiting significant AIE behavior. The double bond between electroactive units is prone to nucleophilic addition reactions, which hinders the intramolecular charge transfer (ICT) processes and, thus, the turning off of fluorescence properties, leading to an efficient probe for cyanide ions.

## 2. Results

### 2.1. Molecular Design, Synthesis, and Structural Characterization

**Scheme 1:**
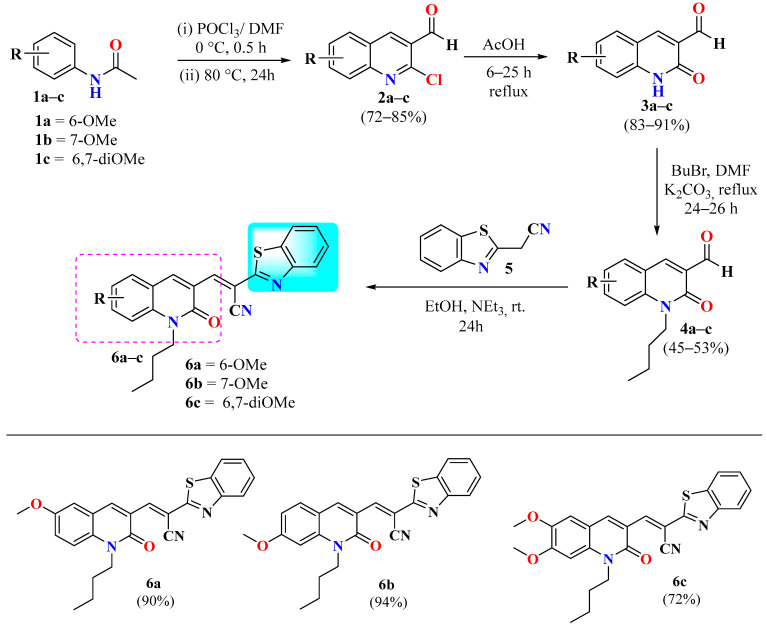
Synthesis and structures of compounds **6a–c**.

The synthesized fluorescent hybrids are based on quinolones and benzothiazole moieties linked through a vinyl group (Scheme 1). A CN group attached to the vinyl fragment as part of a conjugated electron donor–acceptor system (**6a–c**) makes the system more prone to nucleophilic addition reactions of CN^−^ ions to the hybrid system, creating an efficient sensor towards CN^−^ ions. The β-carbon of the vinyl group is electron deficient, making this carbon susceptible to the cyanide attack, triggering a cut in the electronic communication of the hybrid system and, thus, generating remarkable optical and electronic changes used to detect the presence of this ion. Compounds (*E*)-2-(benzo[d]thiazol-2-yl)-3-(2-oxo-1,2-dihydroquinolin)acrylonitriles **6a–c** are shown in Scheme 1. These compounds were synthesized through a classic Knoevenagel condensation [44,45], from stirring equimolar amounts of compounds **4a–c**, benzothiazole **5** and three drops of NEt_3_ in 25 mL of EtOH at room temperature for 48 h. The reaction was controlled by TLC using dichloromethane as eluent, and, once it finished, it was filtered and washed with EtOH thoroughly, giving rise to the respective compounds **6a–c** with yields between 60% and 71%. The structures of these new fluorescent hybrids were confirmed by ^1^H-NMR, ^13^C-NMR, mass spectrometry, and FTIR, and are shown in the experimental section.

**Figure 1:**
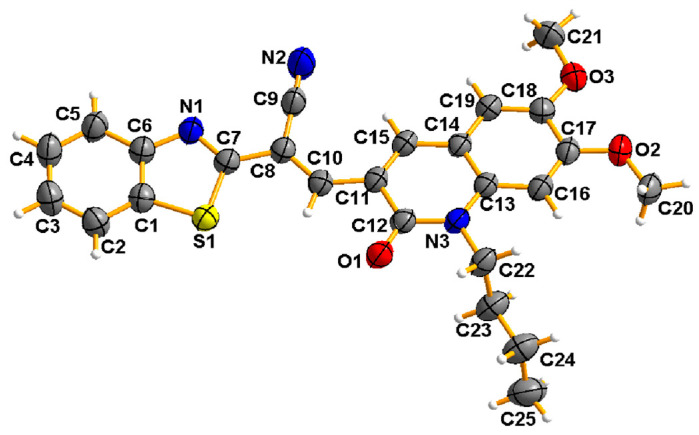
Ellipsoid diagram representation with 50% displacement probability for compound **6c.**

In addition to the aforementioned spectroscopic experiments, and to conclusively establish the formation of target compounds, single crystals of compound **6c** were obtained by slow evaporation in DMF at room temperature. The structural representation, crystal data, and structure refinement for compound **6c** are presented in Figure 1 and the Supporting Information. Based on the results, there is unequivocal evidence that the compound obtained corresponds to product **6c**.

**Figure 2:**
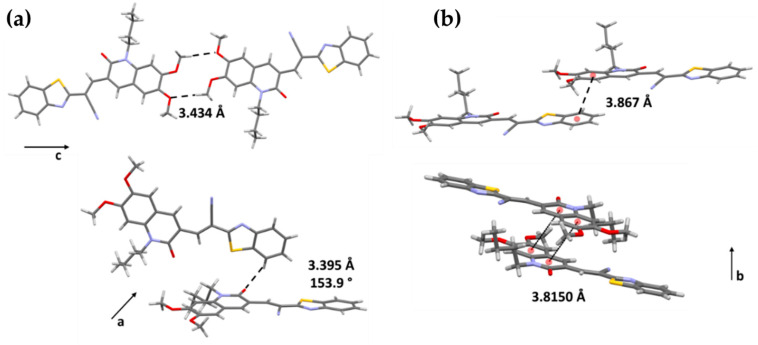
Supramolecular (**a**) C-H∙∙∙O and (**b**) π∙∙∙π interactions presented in the crystal packing of compound **6c**. The C∙∙∙O and π∙∙∙π distances are shown.

Compound **6c** was obtained as a red crystalline solid. This compound crystallizes in a monoclinic P2_1_/c space group, with one molecule of **6c** by asymmetric unit. Benzothiazole and quinolone residues present a small planarity deviation with a torsion angle between them of 8.27(9) Å. The crystal packing of this compound is mainly formed by C-H∙∙∙O and slipped π∙∙∙π stacking interactions (Figure 2). Each molecule forms a R^2^_2_(12) heterosynthon between methoxy groups through C-H∙∙∙O interactions oriented along the [001] direction, with C∙∙∙O distances of 3.434(3) Å (symmetry code: −x, 1−y, 2−z). The dimeric formation is stacked along [010] direction by slipped π∙∙∙π stacking interactions with intercentroid distances of 3.8671(12) and 3.8150(11) Å (symmetry codes: x, y, 1+z and −x, 1−y, 1−z) (Table S1). Also, it is possible to observe C-H∙∙∙O interactions along the [100] direction with a C∙∙∙O distance of 3.395(3) Å (symmetry code: x, 3/2−y, −1/2+z) (Figure 2).

### 2.2. Photophysical Properties

**Figure 3:**
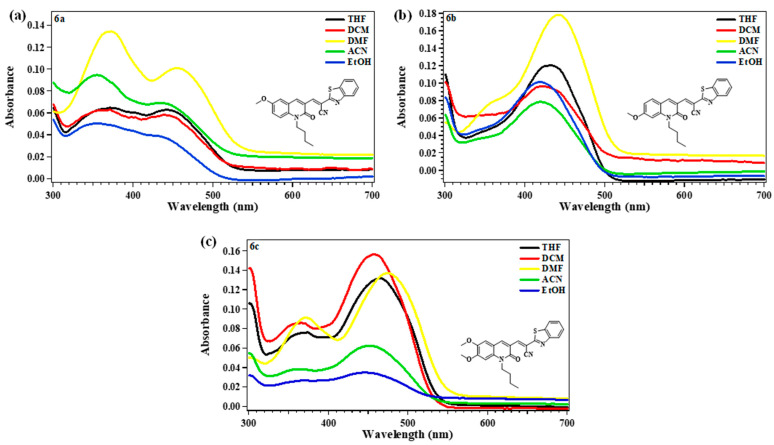
Absorption spectra of (**a**) **6a**, (**b**) **6b,** and (**c**) **6c** in different solvents (THF, DCM, DMF, ACN and EtOH).

The photophysical properties of compounds **6a–c** were evaluated by recording the absorption and emission spectra in five different solvents (THF, DCM, DMF, ACN, and EtOH) at a concentration of 5μM, as summarized in Table 1. The UV-Vis absorption spectra of compounds **6a–c** (Figure 3) showed two leading bands; the first band, centered around 360 nm, can be assigned to π – π* transitions, while the second band, centered in the region of 431–473 nm, can feasibly be attributed to intramolecular charge transfer (ICT) processes [46,47]. Theoretical calculations were carried out using time-dependent uwb97xd/6-311^++^g (d,p) level in THF to find out the nature of the electronic transitions responsible for the observed experimental absorption bands. The intense absorption band experimentally registered around 431–473 nm corresponds to the transition of the lowest-lying singlet excited state S_1_ predicted with a considerable oscillator strength (f = 0.7026, 1.1883, and 1.0113 for **6a**, **6b**, and **6c**, respectively). The transition displayed by the HOMO→LUMO mono-excitation is present in all the dyes.

**Figure 4:**
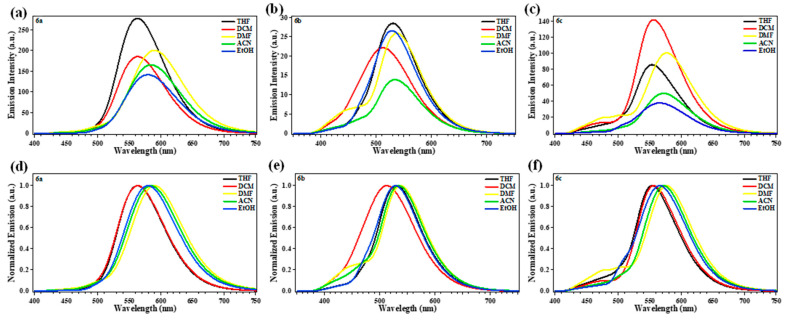
Emission (**a**–**c**) and normalized emission spectra (**d**–**f**) of compounds **6a–c**.

The fluorescence emission spectra of compounds **6a–c** (Figure 4) showed maximum emission bands between 512 and 590 nm. All the compounds exhibited broad Stokes shifts (**6a** 4913–5971, **6b** 3932–5069, and **6c** 3468–4789 cm^−1^) in different solvents, indicating a fast relaxation from the excited state to lower energy vibrational states, which can avoid the interference caused by the excitation light and scattered light, as well as the fluorescence intensity that is diminished by self-absorption, as a result improving the response sensitivity of fluorescent molecules [48]. As the polarity solvents change from THF to EtOH, the emission peaks of **6a**, **6b**, and **6c** showed a bathochromic behavior from 563 to 578, 529 to 528, and 553 to 565 nm, respectively. However, all the chromophores presented a hypsochromic effect in solvents such as DMF, ACN, and EtOH (Figure 4). These findings suggest that the bathochromic effect is due to the ICT state being significantly more polar than the ground state, leading to stronger interactions with polar solvents in the ICT state (Figure S2). On the other hand, the hypsochromic effect is attributed to the difference in solvation since EtOH is an HBD solvent that can stabilize the ground state through H-bonding formation with the methoxy and carbonyl groups of the chromophores [49]. Solvatochromic behavior was studied through solvent polarity plots such as Lippert–Mataga and McRae plots (Figure S1) [50,51]. Lippert–Mataga and McRae show similar trends with a moderate linear relationship for compounds **6a** and **6b**, while compound **6b** exhibits a poor linear tendency. This behavior may be attributed to specific solvent effects, such as preferential solvation, hydrogen bonding, and charge–transfer interactions, which are influenced by one or a few neighboring molecules and governed by the diverse chemical properties of the fluorophore and solvent. Additionally, the optical band gap (E_0-0_) was determined by the interception of the absorption and emission bands for all the compounds, yielding values of 2.41, 2.58, and 2.40 eV for **6a**, **6b**, and **6c**, respectively, exhibiting the same trend as the calculated energy gap in THF.

### 2.3. Aggregation-Induced Emission

**Figure 5:**
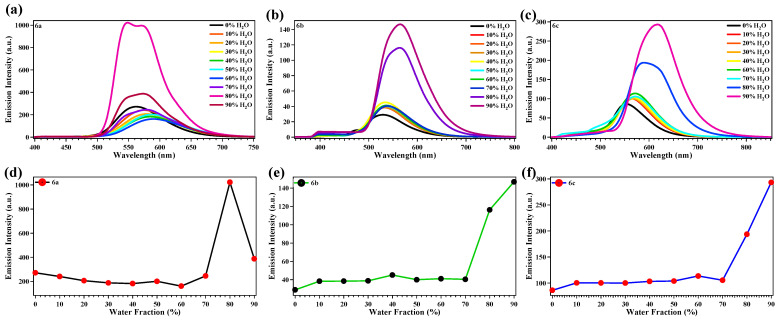
Emission spectra of compounds (**a**) **6a**, (**b**) **6b,** and (**c**) **6c,** and plots of emission intensity vs. water fraction (fw%), (**d**) **6a**, (**e**) **6b**, and (**f**) **6c,** in THF/H_2_O mixtures from 0 up to 90% of water.

To investigate the effects of aggregation on the luminescence behavior of the synthesized compounds, emission spectra of **6a–c** were registered in different THF–water mixtures (f_w_ = 0 to 90%) (Figure 5). Compound **6a** showed a fluorescence quenching with a red shift upon increasing amounts of water (f_w_) until 60% was reached, attributed to the influence of the TICT effect [52,53]. Furthermore, it is acknowledged that when dyes aggregate, they can create π-π stacked structures of chromophores. Ultimately, this leads to a reduction in the band gap and a movement towards longer wavelengths in the emission peaks [54]. Upon increasing the water fraction from 70 to 80%, the compound exhibits a striking fluorescence enhancement with a blue shift. This could be attributed to the formation of aggregates due to the reduced ability of the aqueous mixture to dissolve substances. Above 90% water fraction, the fluorescence emission rapidly diminished.

On the other hand, for compounds **6b** and **6c**, a similar trend was observed. The fluorescence was quenched in both compounds with a bathochromic effect when the water fraction increased up to 70%. After increasing the amount of water from 70 to 90%, the compounds displayed an enhancement in emissions with a red-shift, probably because of the aggregation of dyes leading to the formation of π-π stacked structures of the fluorophores, resulting in a reduced band gap [54]. It is important to note that when large aggregates form at higher f_w_, the twisting motion of the molecular subunits becomes restricted, thus interrupting the TICT process.

### 2.4. Sensing Response of ***6a–c*** to Several Anions

**Figure 6:**
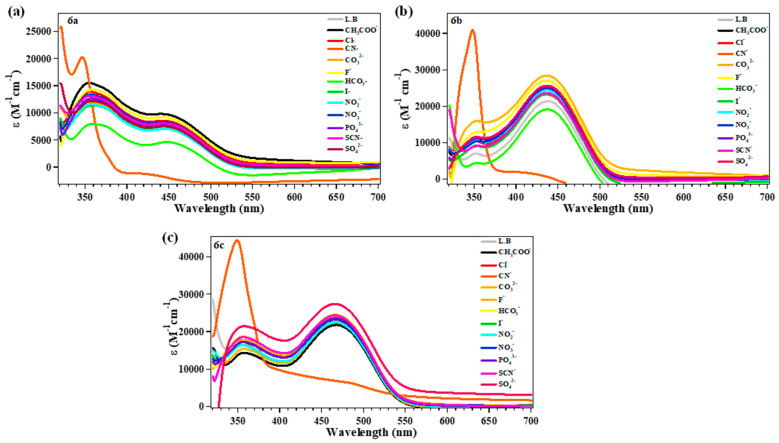
Absorption spectra of (**a**) **6a**, (**b**) **6b,** and (**c**) **6c** in a mixture of DMSO/water of 8:2 at 50 µM with 10 eq of ions CH_3_CO_2_^−^, Cl^−^, CN^−^, CO_3_^2−^, PO_4_^3−^, I^−^, F^−^, NO_2_^−^, NO_3_^−^, HCO_3_^−^, SCN, and SO_4_^2−^ in a pH 7 buffer solution.

The sensing response of quinolone–benzothiazole derivatives toward different anions (CH_3_CO_2_^−^, Cl^−^, CN^−^, CO_3_^2−^, PO_4_^3−^, I^−^, F^−^, NO_2_^−^, NO_3_^−^, HCO_3_^−^, SCN^−^ and SO_4_^2−^) was studied, employing UV-Vis absorption and fluorescence emission measurements in mixtures of DMSO:water (8:2) at a concentration of 50 µM, with 10 equivalents of each anion in a buffer solution at pH 7. Compounds **6a–c** in DMSO/water solution (Figure 6) exhibited two absorption bands around 350 nm and 450 nm, which could be assigned to π-π transitions and the ICT process, respectively. After the addition of 10 equivalents of each of the analytes mentioned above to **6a–c** solutions, no appreciable changes were observed; nevertheless, the addition of CN^−^ anions caused a considerable fall in the absorption intensity at around 450 nm, and the increase in the absorption intensity of the band centered at 350 nm. All these variations can be noted by a change in the color of the solution of compounds **6a–c** once CN^−^ was added, passing from orange to transparent.

**Figure 7:**
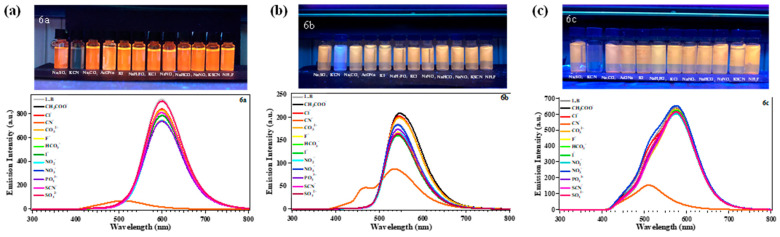
Emission spectra of (**a**) **6a**, (**b**) **6b,** and (**c**) **6c** in a mixture of DMSO/water 8:2 at 50 µM with 10 eq of ions CH_3_CO_2_^−^, Cl^−^, CN^−^, CO_3_^2−^, PO_4_^3−^, I^−^, F^−^, NO_2_^−^, NO_3_^−^, HCO_3_^−^, SCN y SO_4_^2−^ in a pH 7 buffer solution.

**Figure 8:**
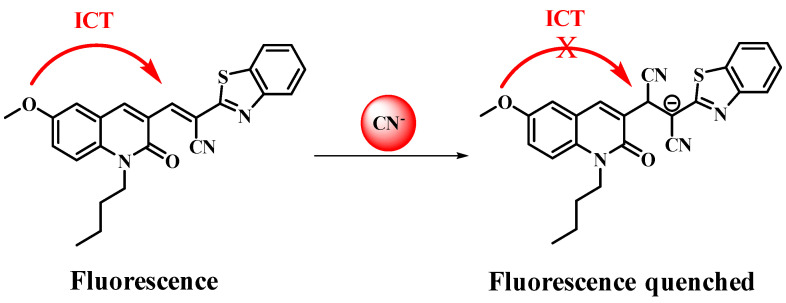
Nucleophilic addition reaction of compound **6b** in presence of cyanide ion.

**Figure 9:**
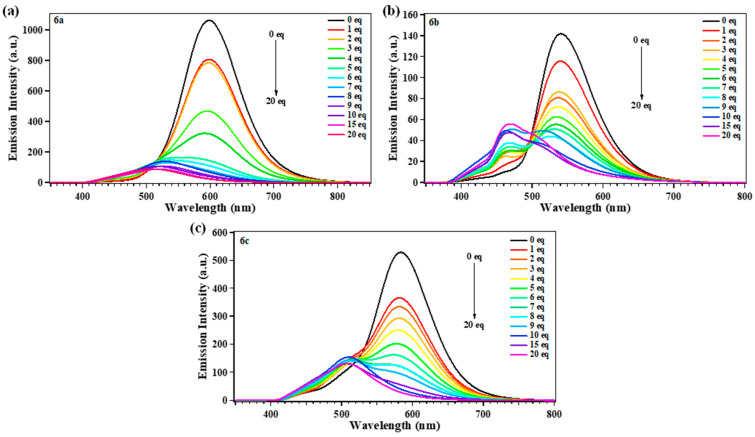
Fluorescence titration spectra of (**a**) **6a**, (**b**) **6b,** and (**c**) **6c** upon the increasing amounts of CN^−^ (0–20 equiv.).

Fluorescence spectra of compounds **6a–c** (Figure 7) show the response against different anions. No anion evidence had a substantial effect on the fluorescence intensity of the compounds **6a–c**, except for the CN^−^ anion, which led to a quenching of the emission peak at 600 nm, 555 nm, and 575 nm for **6a**, **6b**, and **6c**, respectively, with the increase in intensity of peak around 500 nm for **6a** and **6c**. The case of **6b** exhibited two new emission peaks centered at 455 nm and 555 nm. The decrease in emission intensity is probably due to the nucleophilic addition of the cyanide ion in the unsaturation that binds quinolone and benzothiazole units in compounds **6a–c**, inhibiting intramolecular charge transfer, thus quenching fluorescence [55] (Figure 8). The sensing performances of **6a–c** to different amounts of CN^−^ in DMSO/water (8:2) solutions was studied by fluorescence emission spectroscopy. As seen in Figure 9, with the addition of CN^−^, the peaks at 563 nm, 529 nm, and 553 nm for **6a**, **6b,** and **6c,** respectively, gradually decreased because the nucleophilic attack of CN^−^ interrupted the conjugation of the chemosensors, interrupting the ICT process. It is worth highlighting that the gradual increase in the CN^−^ concentration showed an excellent linear relationship (**6a** R^2^: 0.951, **6b** R^2^: 0.962, and **6c** R^2^: 0.902). However, when the concentration of CN^−^ is above 300 µM, the fluorescence intensity remains almost constant, meaning the solution is saturated with CN^−^ ions. According to the equations LOD = 3σ/S and LOQ = 10σ/S, the limit of detection (LOD) and limit of quantification (LOQ) were determined for all the chemosensors (Figure S3). Estimated LOD and LOQ values were, respectively, 3.0 × 10^−7^ M and 9.2 × 10^−7^ M for **6a**, 2.9 × 10^−7^ M and 8.8 × 10^−7^ M for **6b**, and 4.4 × 10^−7^ M and 1.3 × 10^−6^ M for **6c**, which are lower than the allowed amount of CN^−^ in drinking water (1.9 µM) according to the WHO [56].

### 2.5. Selectivity and Competition Experiments for CN^−^

**Figure 10:**
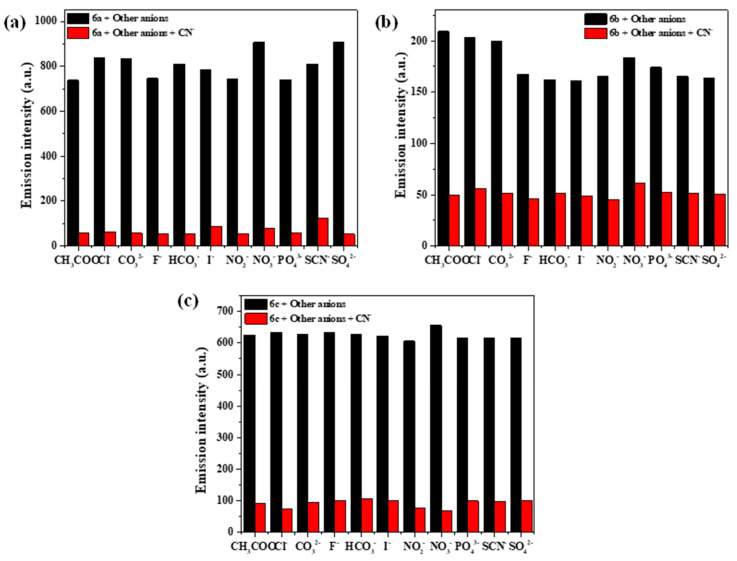
Selectivity test of probes (**a**) **6a**, (**b**) **6b,** and (**c**) **6c** in a DMSO:water mixture 8:2 at 50 µM with 10 eq of ions CH_3_CO^2−^, Cl^−^, CO_3_^2−^, PO_4_^3−^, I^−^, F^−^, NO_2_^−^, NO_3_^−^, HCO_3_^−^, SCN, and SO_4_^2−^ black bar and with CN^−^ ion red bar, in buffer solutions, at pH 7.

To further explore the utility of **6a**, **6b**, and **6c** as an ion-selective fluorescent sensor for CN^−^, competitive studies were carried out with 10 equivalents of CH_3_CO^2−^, Cl^−^, CO_3_^2−^, PO_4_^3−^, I^−^, F^−^, NO^2−^, NO^3−^, HCO^3−^, SCN, and SO_4_^2−^ anions in DMSO/water mixture (8:2). As shown in Figure 10, the introduction of interfering anions to **6a**, **6b,** and **6c** solutions with CN^−^ did not cause significant effects on the fluorescence response. Compounds **6a** and **6c** show better results, indicating that they could be used as a fluorescence sensor for CN^−^ detection with outstanding selectivity and anti-interference.

As shown in Figure 7, the decrease in fluorescence intensity is maximal for CN^−^ ions. In this sense, to have more insight into this fact, a Density Functional Theory (DFT) study was carried out using the combination of the correlation-exchange functional wB97XD, the 6-31G (++dp) basis set, and the frontier orbitals. On the other hand, the solvent effect was studied using the conductor-like polarizable continuum model (CPCM). The application of CPCM extends to the computation of aqueous solvation-free energies for various organic molecules and the elucidation of fluorescence phenomena exhibited by certain dyes. Therefore, the combination of DFT jointly with CPCM could allow us to obtain a better understanding of the fluorescence behavior.

**Figure 11:**
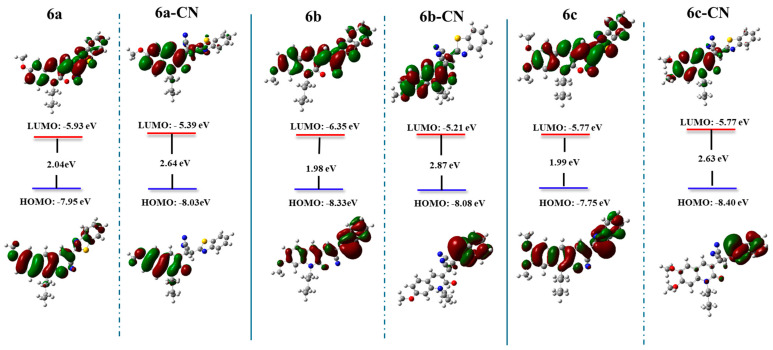
Optimal structures and electronic densities of HOMO and LUMO for compounds **6a–c** (left) and the **6a–c** + CN^−^ system (right).

Figure 11 shows that, as expected, molecules **6a**, **6b,** and **6c** have both HOMO and LUMO spread almost throughout the molecules, which explains the electronic delocalization around the entire structures, and therefore the respective ICT processes take place in all molecules [57]. Interestingly, for **6a–c** + CN^−^ systems (Figure 11—right), both HOMO and LUMO electronic densities are mainly located only on one side of the molecules; for example, for compound **6a**, the HOMO and LUMO are localized over the quinolone moiety, whereas **6b**–CN and **6c**–CN have the HOMO on the benzothiazole moiety and the LUMO over the quinoline moiety. These results strongly suggest that the electronic communication between the quinolone and benzothiazole moieties has been disrupted by the binding of the CN group to the molecule, thus hindering the ICT processes in the molecule; therefore, the fluorescence emission is decreased.

## 3. Materials and Methods

### 3.1. Materials and Instrumentation

All organic chemicals and solvents were purchased from Sigma-Aldrich, Fluka, and Merck (analytical grade reagents) and used without further purification. IR spectra were recorded on a Shimadzu FTIR 8400 ATR spectrophotometer. Melting points were measured using a Stuart SMP3 melting point apparatus and were uncorrected. ^1^H and ^13^C NMR spectra were recorded on a Bruker Advance 400 spectrophotometer (Bremen, Germany) operating at 400 MHz and 100 MHz, respectively, using DMSO-*d*_6_ and CDCl_3_ as solvents and tetramethylsilane as internal standard. ^1^H and ^13^C NMR spectra of all compounds can be found in the supporting information (Figures S4–S6). Mass spectra were run on a SHIMADZU-GCMS 2010-DI-2010 spectrometer (Kyoto, Japan) (equipped with a direct inlet probe) operating at 70 eV. TLC analyses were performed on silica gel aluminum plates (Merck 60 F254), and spots were visualized with ultra-violet irradiation. Single crystal X-ray diffraction data were collected at 298 K on an Agilent SuperNova, Dual, Cu at Zero, Atlas four-circle diffractometer equipped with a CCD plate detector using CuKα radiation (λ = 1.54184 Å). Data integration, Lorentz polarization effects, and absorption corrections were performed using the CrysAlis PRO software package [58]. The structure was solved by using an iterative algorithm and then refined by SHELXL-2015 [59], included in Olex2 [60]. Non-hydrogen atoms of the molecules were resolved, and their full-matrix least-squares refinement with anisotropic thermal parameters was conducted. Aromatic and aliphatic hydrogen atoms were stereochemically positioned and refined with the riding model (with Uiso(H) = 1.2 Ueq for CH, CH_2_ and CH_3_). The anisotropic displacement ellipsoid plots and artwork were built with Diamond [61] and Mercury [62] software packages. The CIF file of compound 6c was deposited in the Cambridge Structural Data Base under the code CCDC 2341851. Copies of the data can be obtained, free of charge, via www.ccdc.cam.ac.uk (accessed on 27 June of 2024). Density Functional Theory (DFT) calculations for pristine **6a–c** compounds and **6a–c** + CN⁻ systems were performed using the B3LYP/6-31G(d) level of theory. These calculations were carried out with the Gaussian 09 (G09) software package, specifically the Linux version. The computational work was executed on a workstation equipped with 64 cores and 125 GB of RAM.

### 3.2. General Procedure for the Synthesis of ***2a–c*** and ***3a–c***

*N*,*N*-dimethylformamide (9.1 g, 9.6 mL, 0.125 mol) was cooled to 0 °C and phosphoryl chloride (53.7 g, 32.2 mL, 0.35 mol) was added dropwise, while stirring, for 30 min. The corresponding acetanilide **1a–c** (0.05 mol) was added to this solution and the temperature was raised to 80 °C for 24 h. The cooled reaction mixture was poured into ice water (300 mL) and stirred at 0–10 °C for 1 h. The resulting precipitate was filtered, washed with water (100 mL), dried, and recrystallized from ethyl acetate to give products **2a–c**. Subsequently, to obtain intermediates **3a–c**, aldehydes **2a–c** (1 mmol) were refluxed in 10 mL of 70% aqueous acetic acid for 6–25 h. After cooling, the resulting solid precipitate was collected by filtration, washed with water, dried, and purified by recrystallization from *N*,*N*-dimethylformamide (DMF).

### 3.3. General Procedure for the Synthesis of ***5a–c***

1-Bromobutane (1.3 mmol) and potassium carbonate (1.5 mmol) were added to a solution of quinolones **3a–c** (1.0 mmol) in DMF (4 mL). The reaction mixture was stirred at room temperature for 24–26 h. After completion (checked by TLC), the reaction mixture was poured into ice water (25 mL) and the solid product which was formed was filtered, washed well with water, dried, and purified by column chromatography using dichloromethane.

### 3.4. General Procedure for the Synthesis of Hybrids **6a–c**

Compounds **4a–c** (1.00 mmol) were dissolved in 25 mL of EtOH in a round-bottom flask; compound **5** (1.19 mmol) was then added to the solution, followed by the addition of three drops of tri-ethylamine (NEt_3_). The mixture was stirred for 24 h at room temperature. The reaction was controlled by TLC, and once the reagents were consumed, the mixture was filtered and was washed several times with ethanol. No further purification was required.

(*E*)-2-(Benzo[d]thiazol-2-yl)-3-(1-butyl-6-methoxy-2-oxo-1,2-dihydroquinolin-3-yl)acrylonitrile (**6a**). Yield 90%. Orange solid, m.p. 225–227 °C, FTIR (ATR) υ = [2225] (C≡N), [1637] (C=O) cm^−1^, ^1^H NMR (400 MHz, CDCl_3_) δ (ppm): 8.91 (s, 1H), 8.67 (s, 1H), 8.14 (d, *J* = 8.1 Hz, 1H), 7.92 (d, *J* = 8.0 Hz, 1H), 7.55 (t, *J* = 8.2 Hz, 1H), 7.46 (t, *J* = 7.6 Hz, 1H), 7.37–7.25 (m, 2H), 7.17 (d, *J* = 2.4 Hz, 1H), 4.48–4.26 (m, 2H), 3.93 (s, 3H), 1.86–1.72 (m, 2H), 1.66–1.45 (m, 2H), 1.05 (t, *J* = 7.3 Hz, 3H). ^13^C NMR (101 MHz, CDCl_3_) δ (ppm): 163.1, 160.1, 155.1, 153.7, 141.6, 138.8, 134.9, 134.8, 126.9, 126.3, 124.4, 124.1, 123.1, 121.6, 120.7, 116.0, 115.8, 111.0, 107.6, 55.9, 43.3, 29.7, 20.3, 13.8. EI MS (70 eV): *m/z* 415 (63.6%), 342 (14), 313 (34), 239 (35), 236 (60), 133 (78), 83 (100). HRMS (ESI) *m/z*: [M + H]^+^ calc. for C_24_H_22_N_3_O_2_S 416.1427; found 416.1421.

(*E*)-2-(Benzo[d]thiazol-2-yl)-3-(1-butyl-7-methoxy-2-oxo-1,2-dihydroquinolin-3-yl)acrylonitrile (**6b**), Yield 94%. Orange solid, m.p. 240–241 °C FTIR (ATR) υ = [2218] (C≡N), [1651] (C=O) cm^−1^, ^1^H NMR (400 MHz, CDCl_3_) δ (ppm): 8.92 (s, 1H), 8.65 (s, 1H), 8.13 (d, *J* = 8.0 Hz, 1H), 7.91 (d, *J* = 7.7 Hz, 1H), 7.70 (d, *J* = 8.8 Hz, 1H), 7.54 (t, *J* = 7.7 Hz, 1H), 7.45 (t, *J* = 8.1 Hz, 1H), 6.92 (dd, *J* = 8.7, 2.1 Hz, 1H), 6.82 (d, *J* = 1.8 Hz, 1H), 4.38–4.23 (m, 2H), 3.98 (s, 3H), 1.88–1.73 (m, 2H), 1.67–1.42 (m, 2H), 1.06 (t, *J* = 7.4 Hz, 3H). ^13^C NMR (101 MHz, CDCl_3_) δ (ppm): 164.0, 163.0, 161.0, 153.7, 142.1, 141.8, 139.3, 134.8, 132.9, 126.8, 126.1, 123.9, 121.6, 120.8, 116.3, 114.5, 110.7, 106.0, 99.0, 55.8, 43.2, 29.2, 20.3, 13.9. EI MS (70 eV): *m/z* 414.90 (100%), 384 (23), 373 (21), 358 (34), 342 (53); HRMS (ESI) *m/z*: [M + H]^+^ calc. for C_24_H_22_N_3_O_2_S 416.1427; found 416.1413.

(*E*)-2-(Benzo[*d*]thiazol-2-yl)-3-(1-butyl-6,7-dimethoxy-2-oxo-1,2-dihydroquinolin-3-yl)acrylonitrile (**6c**), Yield 72%. Orange solid, m.p. 243–244 °C FTIR (ATR) υ = [2217] (C≡N), [1630] (C=O) cm^−1^, ^1^H NMR (400 MHz, CDCl_3_) δ (ppm): 8.93 (s, 1H), 8.67 (s, 1H), 8.14 (d, *J* = 8.1 Hz, 1H), 7.92 (d, *J* = 7.9 Hz, 1H), 7.54 (t, *J* = 7.6 Hz, 1H), 7.45 (t, *J* = 7.5 Hz, 1H), 7.13 (s, 1H), 6.80 (s, 1H), 4.47–4.32 (m, 2H), 4.06 (s, 3H), 4.01 (s, 3H), 1.88–1.78 (m, 2H), 1.64–1.44 (m, 2H), 1.08 (t, *J* = 7.4 Hz, 3H). ^13^C NMR (101 MHz, CDCl_3_) δ (ppm): 163.6, 160.6, 154.7, 153.7, 145.9, 141.9, 138.6, 136.8, 134.8, 126.8, 126.1, 123.9, 121.6, 121.1, 116.4, 113.7, 110.6, 105.7, 97.0, 56.3, 43.3, 29.4, 20.4, 13.9. EI MS (70 eV): *m/z* 445 (100%), 428 (26), 414 (24), 388 (36), 372 (54); HRMS (ESI) *m/z*: [M + H]^+^ calc. for C_25_H_24_N_3_O_3_S 446.1538; found 446.1538.

## 4. Conclusions

Three novel aggregation-induced emission (AIE) luminogens based on methoxiquinolone–benzothiazole hybrids were successfully synthesized and characterized. These compounds exhibit a significant increase in emission, accompanied by a red-shifting when the mixture of THF–water contains over 70% water, demonstrating their efficiency as AIE systems. Additionally, these hybrids serve as highly selective and efficient cyanide chemosensors with limits of detection (LOD) and quantifications (LOQ) of 3.0 × 10^−7^ M and 9.2 × 10^−7^ M for compound 6a, 2.9 × 10^−7^ M and 8.8 × 10^−7^ M for compound 6b, and 4.4 × 10^−7^ M and 1.3 × 10^−6^ M for compound 6c. Moreover, the study of frontier molecular orbitals (FMOs) showed that in the absence of cyanide ions (CN^−^), the HOMO and LUMO of compounds **6a–c** are delocalized across the entire molecular structures, facilitating intramolecular charge transfer processes. However, upon the addition of CN^−^, the electronic densities of both HOMO and LUMO become localized on one side of the molecules, indicating a substantial change in electronic distribution and potentially influencing their photophysical behavior. This work paves the way for the design of new AIEgens and efficient cyanide chemosensors based on heterocyclic quinolones and benzothiazole moieties.

## Figures and Tables

**Table 1:** Spectroscopic properties of compounds **6a**, **6b**, and **6c** at 5 µM in different solvents.

Comp.	6a			6b			6c		
Solvents	Abs, nm (ε/M^−1^cm^−1^)	Em, nm	Stokes Shift nm (cm^−1^)	Abs, nm (ε/M^−1^cm^−1^)	Em, nm	Stokes Shift nm (cm^−1^)	Abs, nm (ε/M^−1^cm^−1^)	Em, nm	Stokes Shift nm (cm^−1^)
THF	441 (12,600)	563	122 (4913)	431 (24,140)	529	98 (4298)	464 (26,380)	553	89 (3468)
DCM	438 (11,700)	563	125 (5069)	422 (19,360)	512	90 (4165)	456 (31,340)	556	100 (3944)
DMF	454 (20,240)	590	136 (5077)	442 (35,720)	535	93 (3932)	473 (27,480)	576	103 (3780)
ACN	433 (13,880)	584	151 (5971)	419 (15,760)	532	113 (5069)	449 (12,500)	572	123 (4789)
EtOH	438 (7620)	578	140 (5530)	418 (15,760)	528	110 (4984)	445 (7040)	565	120 (4772)

## Data Availability

Data are contained within the article.

## Supplementary Materials

The following supporting information can be downloaded at https://www.mdpi.com/article/10.3390/ijms252312896/s1.
