# Supplementary material for: Methoxyquinolone–Benzothiazole Hybrids as New Aggregation-Induced Emission Luminogens and Efficient Fluorescent Chemosensors for Cyanide Ions
Source: Int J Mol Sci. 2024 Nov 30;25(23):12896. doi: 10.3390/ijms252312896 (PMC11641556; doi:10.3390/ijms252312896)
Supplement: Supplementary file 1 [file ijms-25-12896-s001.zip › ijms-3320753-supplementary.pdf]

Supplementary Materials

Methoxyquinolone–Benzothiazole Hybrids as New Aggregation-Induced Emission Luminogens and Efficient Fluorescent Chemosensors for Cyanide Ions

Mario Mutis-Ayala [1], Jorge Trilleras [1], Richard D'Vries [2], Mario A. Macías [3], Alberto Insuasty [4], Rodrigo Abonia [4], Jairo Quiroga [4], Luis A. Illicachi [5], Edgar Márquez [6] and Daniel Insuasty [6,*]

[1] Grupo de Investigación en Compuestos Heterocíclicos, Universidad del Atlántico, Puerto Colombia 081007, Colombia; mmutisayala@mail.uniatlantico.edu.co (M.M.-A.); jorgetrilleras@mail.uniatlantico.edu.co (J.T.)
[2] Grupo de Investigación en Química de Productos Naturales, Departamento de Química, Facultad de Ciencias Naturales, Exactas y de la Educación, Universidad del Cauca, Calle 5 # 4–70, Popayán 190003, Colombia; richard.dvries@unicauca.edu.co
[3] Cristalografía y Química de Materiales, CrisQuimMat, Facultad de Ciencias, Departamento de Química, Universidad de los Andes, Cra. 1 #18a-12, Bogotá, Colombia; ma.maciasl@uniandes.edu.co
[4] Grupo de Investigación de Compuestos Heterocíclicos, Departamento de Química, Universidad del Valle, Calle 13 # 100–00, Cali 760032, Colombia; alberto.insuasty@correounivalle.edu.co (A.I.); rodrigo.abonia@correounivalle.edu.co (R.A.); jairo.quiroga@correounivalle.edu.co (J.Q.)
[5] Grupo de Investigación en Química y Biotecnología, Facultad de Ciencias Básicas, Universidad Santiago de Cali, Calle 5 # 62-00, Cali 760035, Colombia; luis.illicachi00@usc.edu.co
[6] Departamento de Química y Biología, División de Ciencias Básicas, Universidad del Norte, Km 5 vía Puerto Colombia, Barranquilla 081007, Colombia; ebrazon@uninorte.edu.co
* Correspondence: insuastyd@uninorte.edu.co

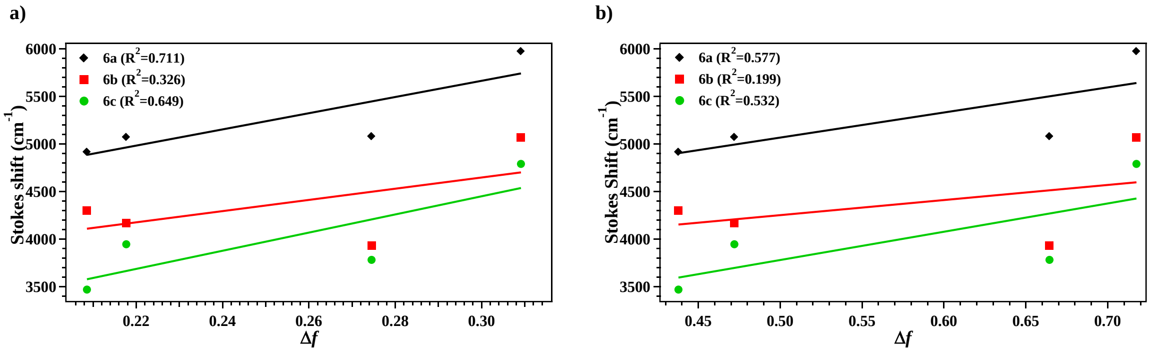

Figure S1. (**a**) Lippert-Mataga and (**b**) McRae plots of chromophores **6a–c**.

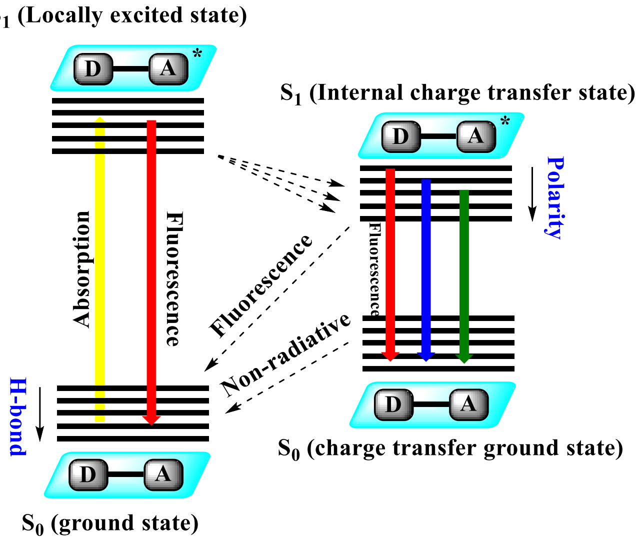

Figure S2. Jablonski diagrams of intermolecular charge transfer.

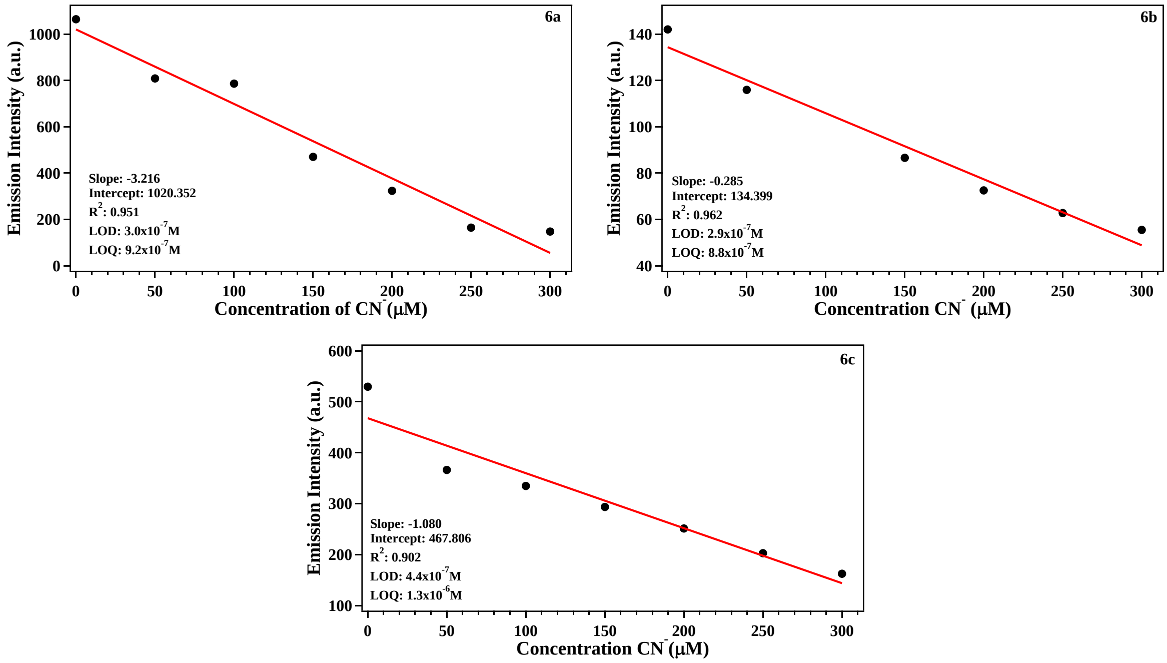

Figure S3. The calibration plot for determination of LOD and LOQ of **6a**, **6b,** and **6c**.

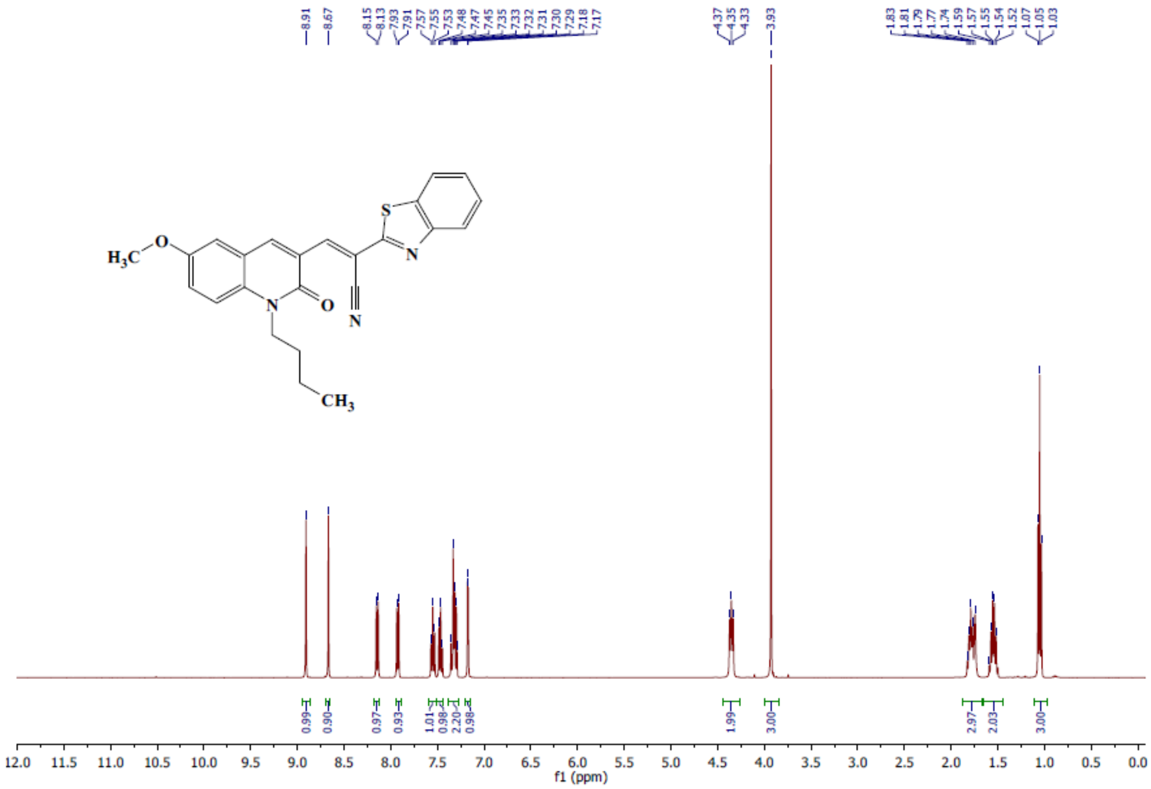

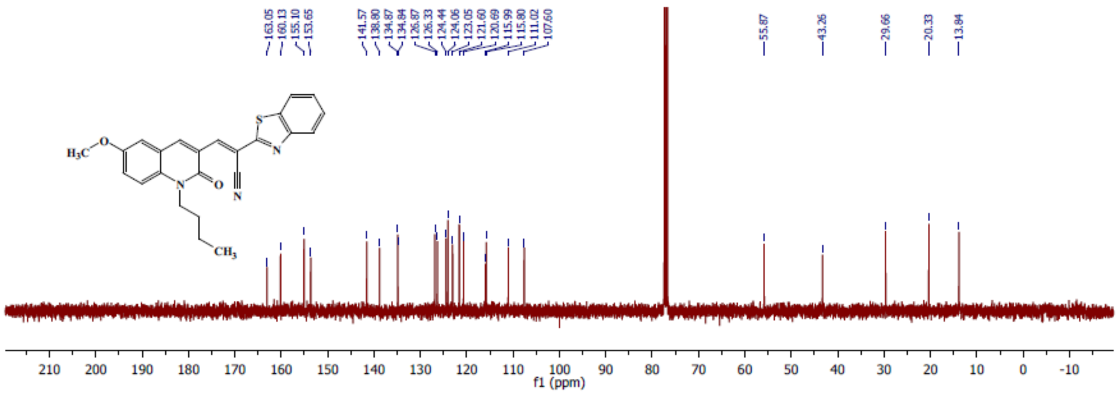

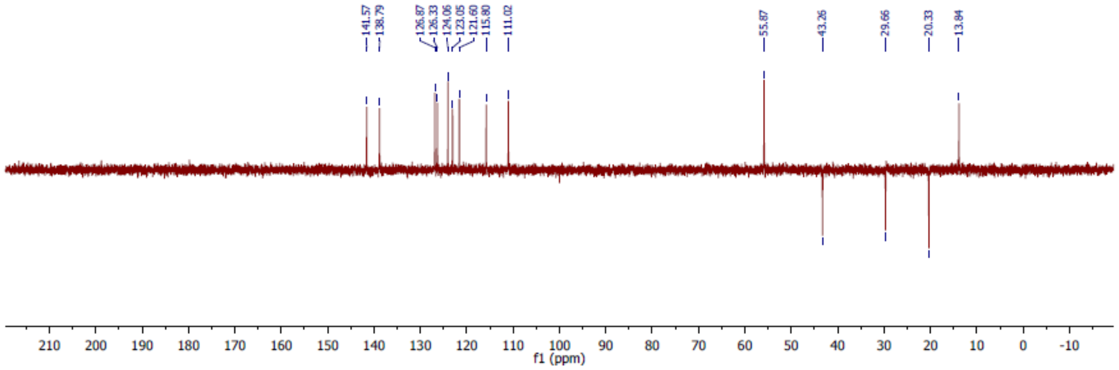

Figure S4. ^{1}H-NMR and ^{13}C-NMR Spectra of compound **6a.**

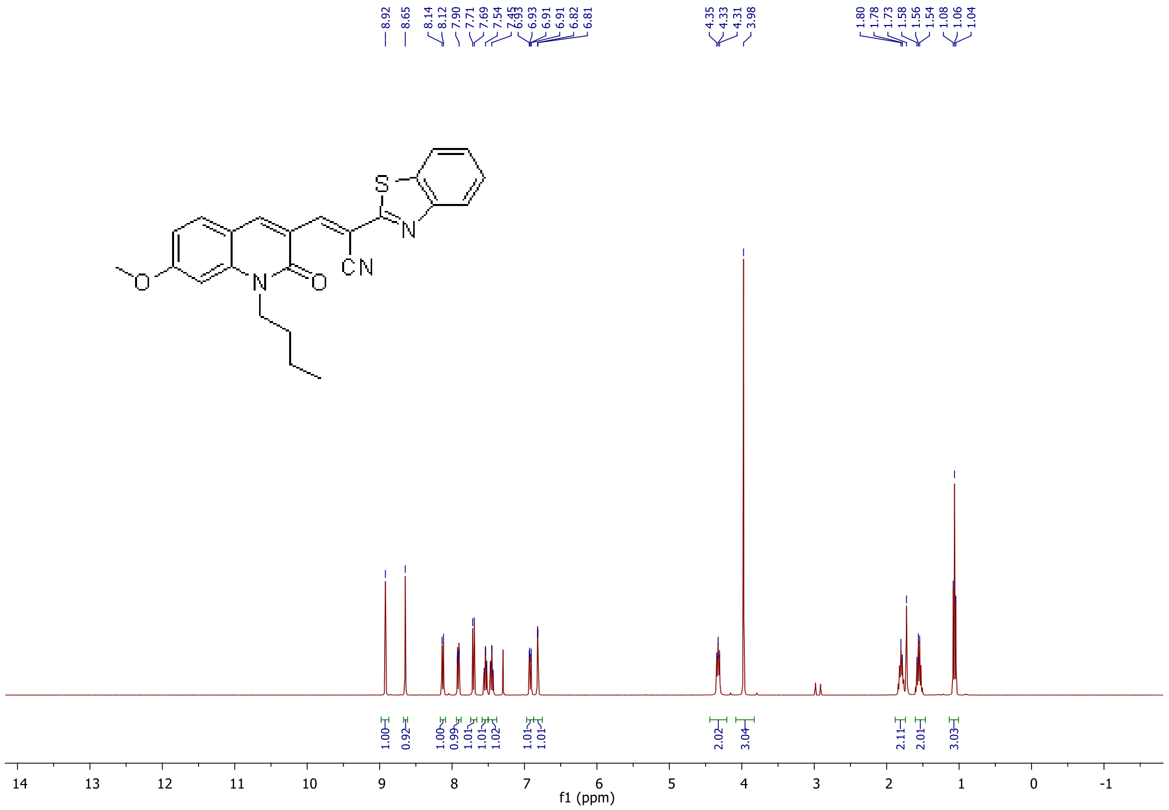

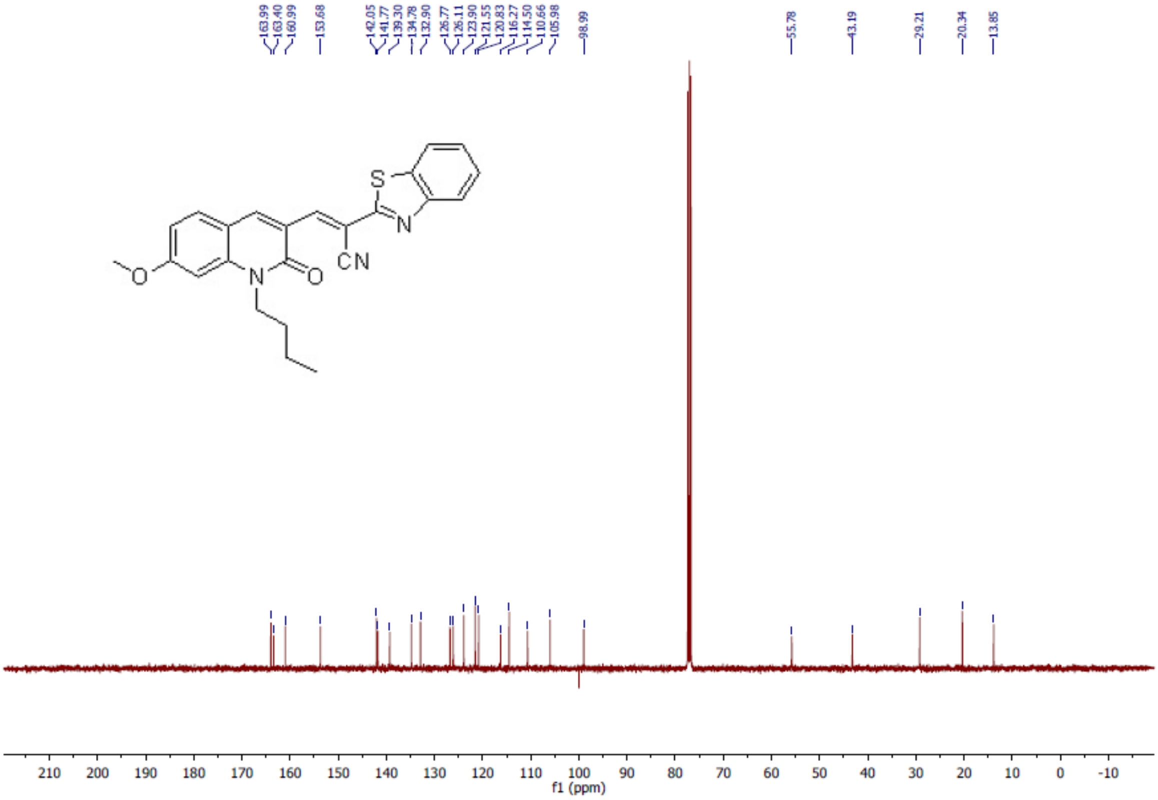

Figure S5. ^{1}H-NMR and ^{13}C-NMR Spectra of compound **6b.**

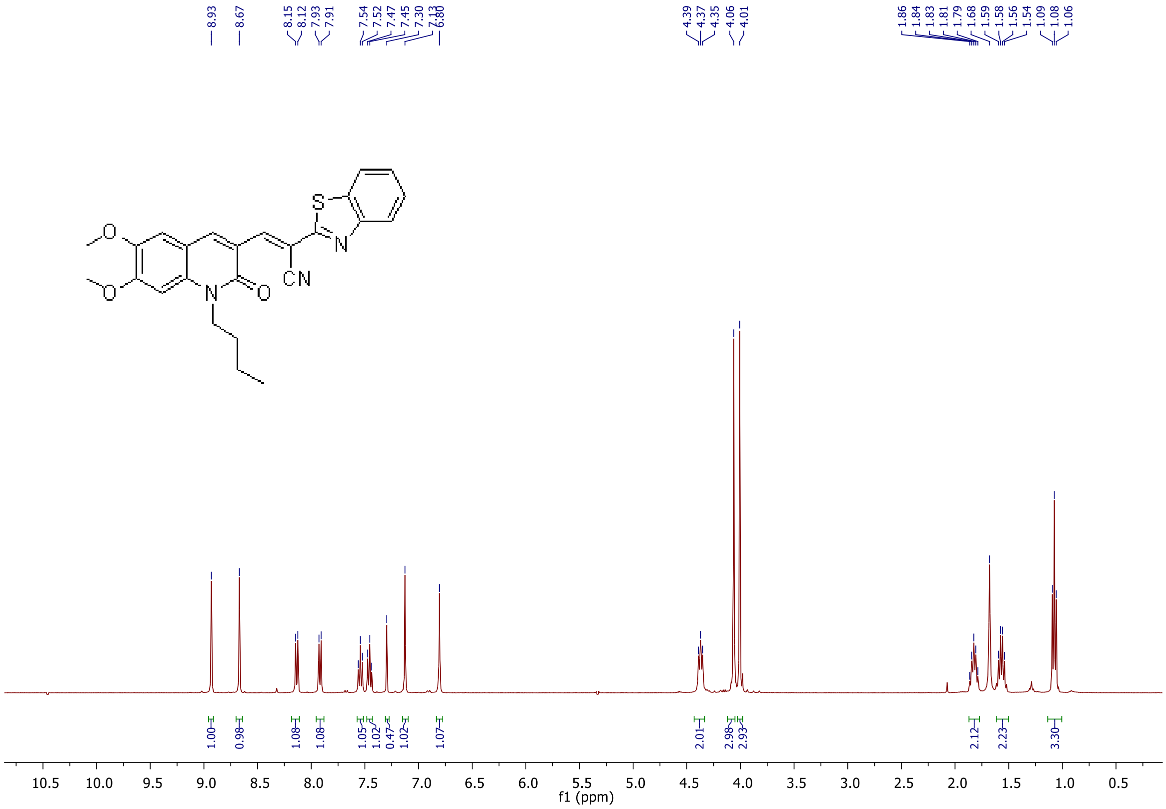

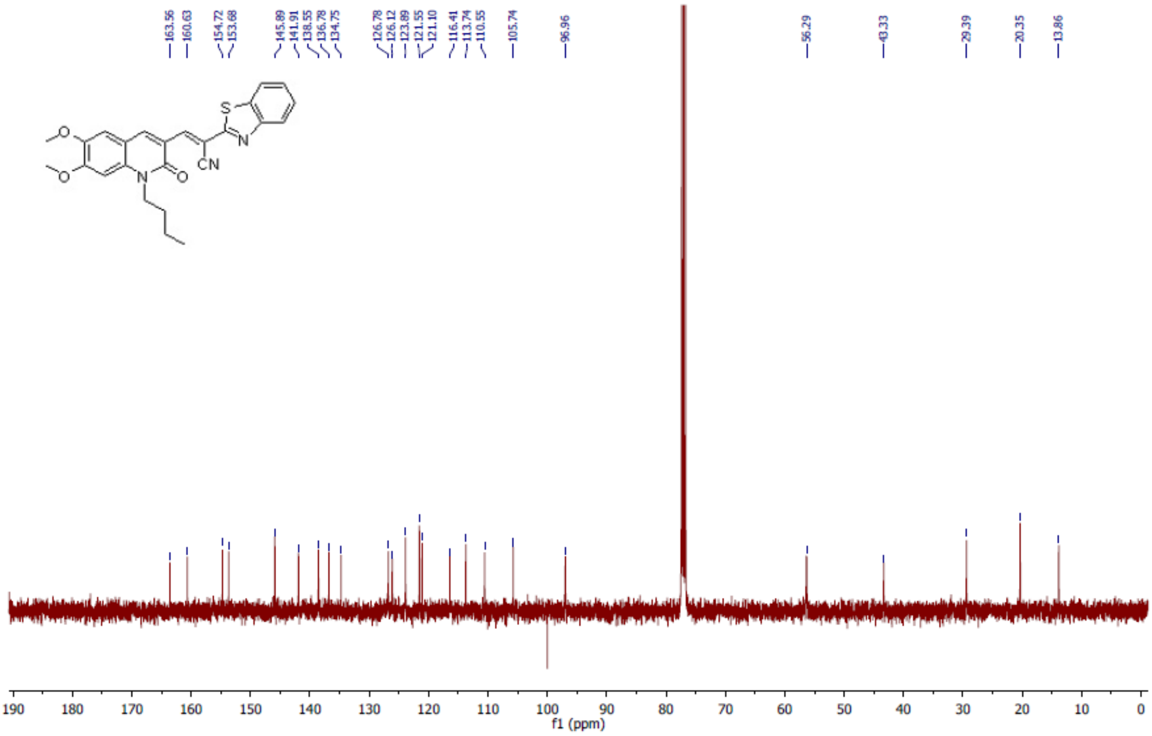

Figure S6. ^{1}H-NMR and ^{13}C-NMR Spectra of compound **6c.**

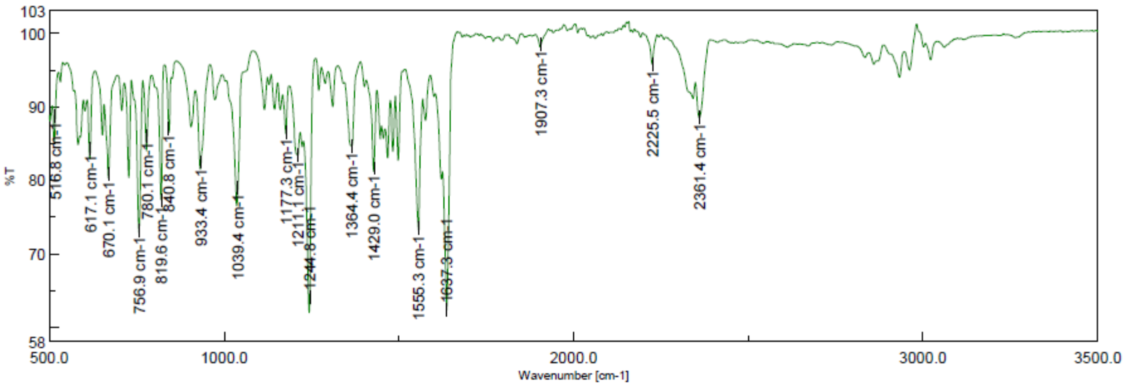

[Comments]
Sample name
Comment
User
Division
Company Universidad Del Atlantico

[Detailed Information]
Creation date 23/04/2024 11:21 a. m.
Date modified 23/04/2024 1:35 p. m.

Data array type Linear data array
Horizontal axis Wavenumber [cm-1]
Vertical axis %T
Start 399.193 cm-1
End 4000.6 cm-1
Data interval 0.964233 cm-1
Data points 3736

[Measurement Information]
Model Name FT/IR-4700typeA
Serial Number F058861788

Accessory ATR PRO ONE
Accessory S/N C179361809
Incident angle 45 deg

Measurement Date 23/04/2024 11:21 a. m.

Light Source Standard
Detector TGS
Accumulation 16
Resolution 4 cm-1
Zero Filling On
Apodization Cosine
Gain Auto (8)
Aperture Auto (7.1 mm)
Scanning Speed Auto (2 mm/sec)
Filter Auto (30000 Hz)

Figure S7. FT-IR Spectra of compound **6a.**

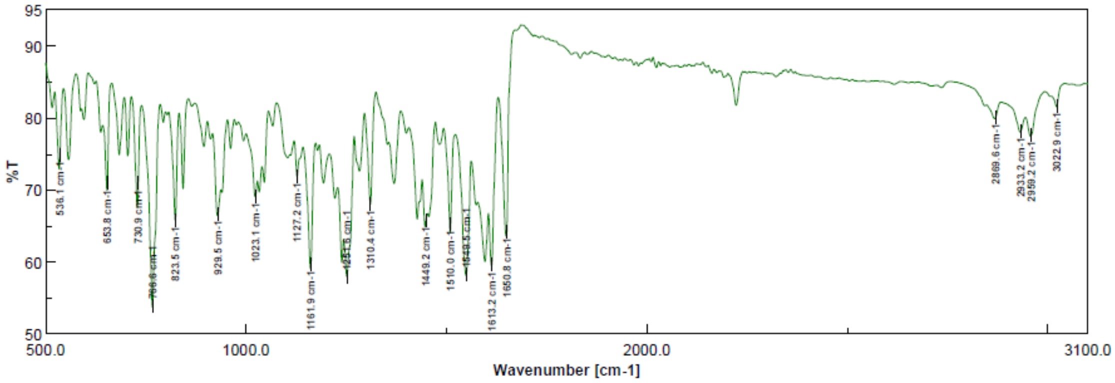

[Comments]
Sample name
Comment
User
Division
Company Universidad Del Atlantico

[Detailed Information]
Creation date 23/04/2024 1:05 p. m.
Date modified 23/04/2024 2:06 p. m.

Data array type Linear data array
Horizontal axis Wavenumber [cm-1]
Vertical axis %T
Start 399.193 cm-1
End 4000.6 cm-1
Data interval 0.964233 cm-1
Data points 3736

[Measurement Information]
Model Name FT/IR-4700typeA
Serial Number F058861788

Accessory ATR PRO ONE
Accessory S/N C179361809
Incident angle 45 deg

Measurement Date 23/04/2024 1:05 p. m.

Light Source Standard
Detector TGS
Accumulation 16
Resolution 4 cm-1
Zero Filling On
Apodization Cosine
Gain Auto (8)
Aperture Auto (7.1 mm)
Scanning Speed Auto (2 mm/sec)
Filter Auto (30000 Hz)

Figure S8. FT-IR Spectra of compound **6b.**

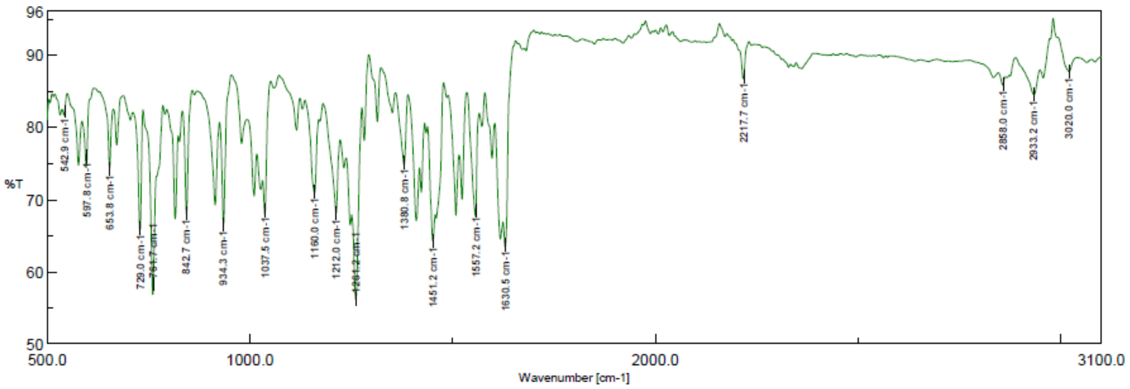

[Comments]
Sample name
Comment
User
Division
Company Universidad Del Atlantico

[Detailed Information]
Creation date 23/04/2024 11:57 a. m.
Date modified 23/04/2024 1:57 p. m.

Data array type Linear data array
Horizontal axis Wavenumber [cm-1]
Vertical axis %T
Start 399.193 cm-1
End 4000.6 cm-1
Data interval 0.964233 cm-1
Data points 3736

[Measurement Information]
Model Name FT/IR-4700typeA
Serial Number F058861788

Accessory ATR PRO ONE
Accessory S/N C179361809
Incident angle 45 deg

Measurement Date 23/04/2024 11:57 a. m.

Light Source Standard
Detector TGS
Accumulation 16
Resolution 4 cm-1
Zero Filling On
Apodization Cosine
Gain Auto (8)
Aperture Auto (7.1 mm)
Scanning Speed Auto (2 mm/sec)
Filter Auto (30000 Hz)

Figure S9. FT-IR Spectra of compound **6c.**

Universidad de los Andes | Facultad de Ciencias
Departamento de Química
QuAnLab

Reporte de Análisis Cualitativo

**Nombre de archivo**	MM-19.d	**Nombre de la muestra**	MM-19
**Tipo de muestra**	Sample	**Posición**	P1-C4
**Instrumento**	G6520B	**Nombre de Usuario**	
**Método Aq**	Default 2021.m	**Fecha de adquisición**	10/16/2024 9:11:55 AM
**IRM Calibration Status**	Success	**Método DA**	Def 2022.m
**Comentario**			

Sample Group **Info.**

User Chromatograms

Fragmentor Voltage 175 **Collision Energy** 0 **Ionization Mode** ESI

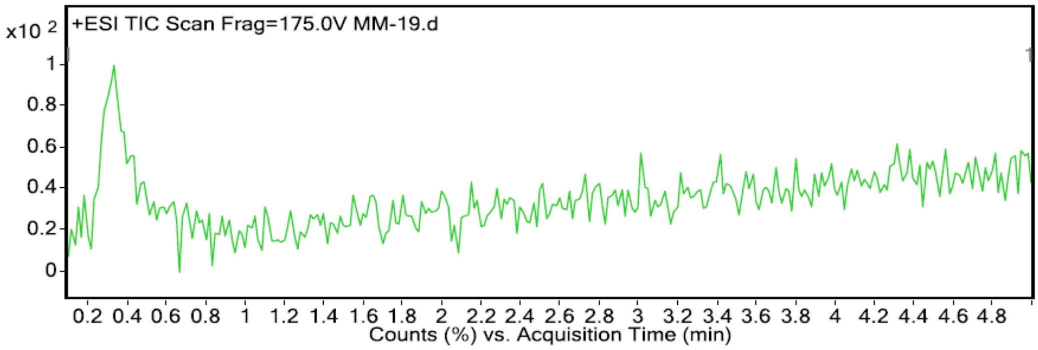

User Spectra

Fragmentor Voltage	Collision Energy	Ionization Mode
175	0	ESI

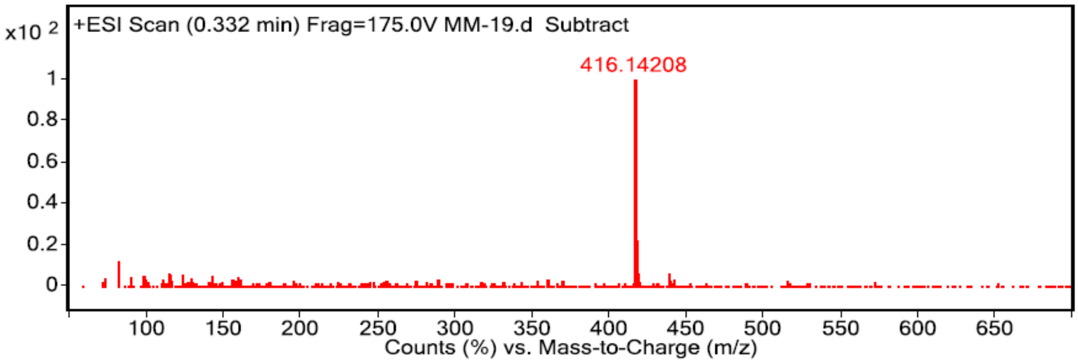

Peak List

m/z	z	Abund	Abund %
81.93788		3728.8	12.27
97.96818		1722.5	5.67
113.96391		1952.8	6.43
114.98515		1737.8	5.72
122.96392		1848.4	6.08
141.95778		1684.5	5.54
416.14208	1	30380.9	100
416.25461		1847.6	6.08

Figure S10. HRMS of compound 6a.

Universidad de los Andes | Facultad de Ciencias, Departamento de Química, QuAnLab

Reporte de Análisis Cualita

**Nombre de archivo**	MM-38.d	**Nombre de la muestra**	MM-38
**Tipo de muestra**	Sample	**Posición**	P1-C6
**Instrumento**	G6520B	**Nombre de Usuario**	
**Método Aq**	Default 2021.m	**Fecha de adquisición**	10/16/2024 9:22:54 AM
**IRM Calibration Status**	Success	**Método DA**	Def 2022.m
**Comentario**			

Sample Group **Info.**

User Chromatograms

Fragmentor Voltage 175 **Collision Energy** 0 **Ionization Mode** ESI

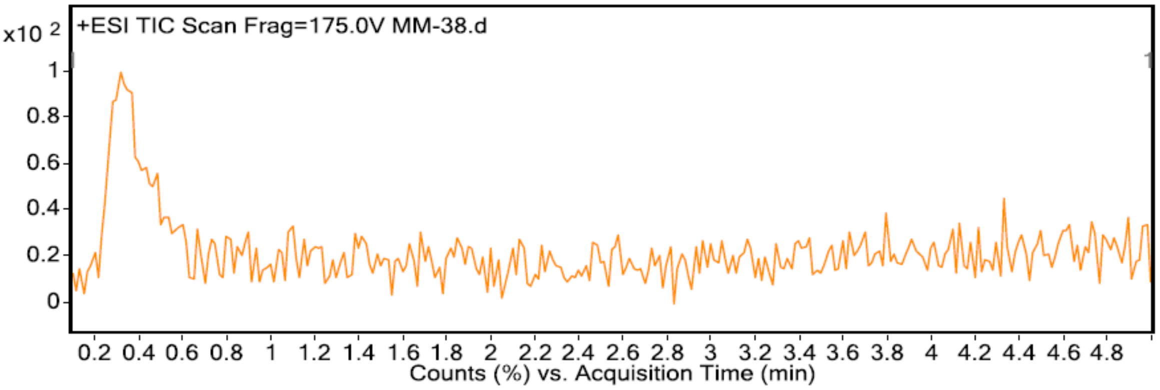

User Spectra

Fragmentor Voltage 175 **Collision Energy** 0 **Ionization Mode** ESI

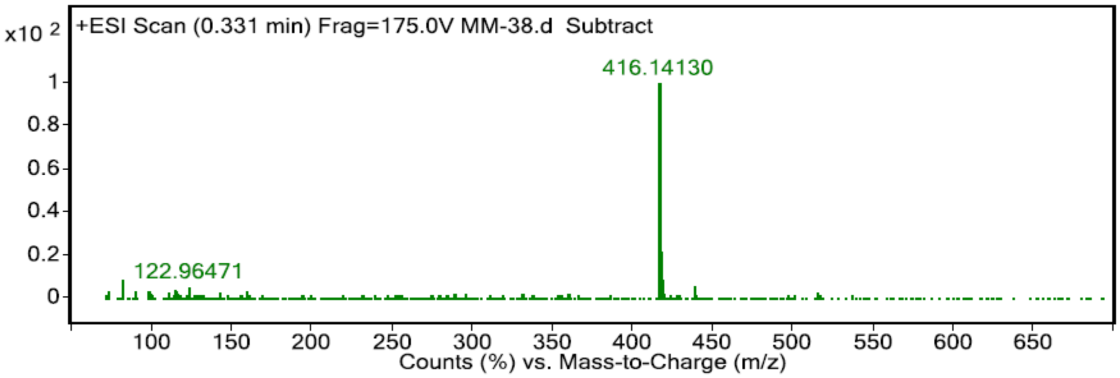

Peak List

m/z	z	Abund	Abund %
81.9379		3702.9	8.46
122.96471		2464.6	5.63
416.1413	1	43755.8	100
416.27933		2281.1	5.21
417.14569	1	9409.3	21.5
418.14222	1	3714.7	8.49
438.12681		2578.4	5.89

Figure S11. HRMS of compound **6b.**

Reporte de Análisis Cualitativo

Universidad de los Andes
Facultad de Ciencias
Departamento de Química
QuAnLab

**Nombre de archivo**	MM 35.d	**Nombre de la muestra**	MM 35
**Tipo de muestra**	Sample	**Posición**	P1-E4
**Instrumento**	G6520B	**Nombre de Usuario**	
**Método Aq**	Default 2021.m	**Fecha de adquisición**	11/1/2024 11:39:51 AM
**IRM Calibration Status**	Success	**Método DA**	Def 2022.m
**Comentario**			

Sample Group **Info.**

User Chromatograms

Fragmentor Voltage 175 **Collision Energy** 0 **Ionization Mode** ESI

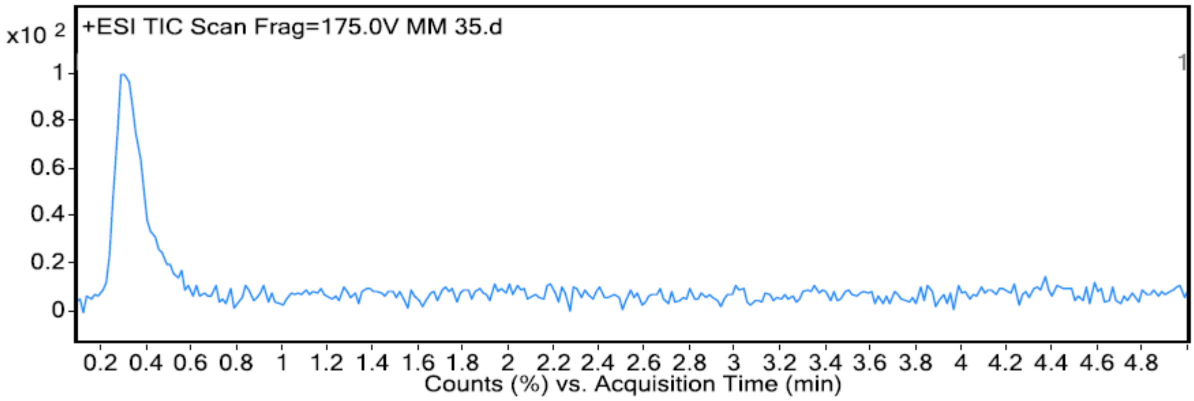

User Spectra

**Fragmentor Voltage**	**Collision Energy**	**Ionization Mode**
175	0	ESI

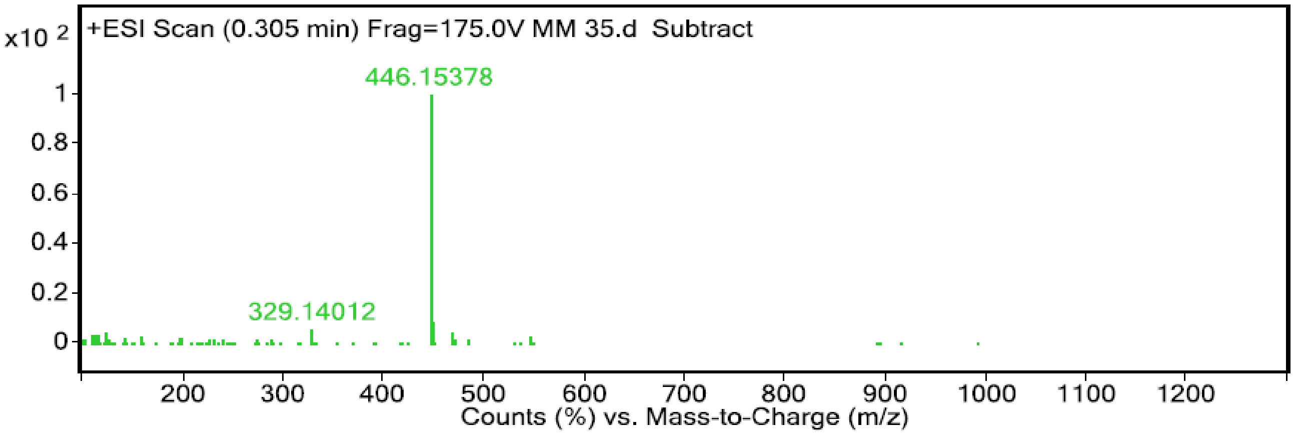

Peak List

m/z	z	Abund	Abund %
81.93819		6827.2	10.33
329.14012		3829.2	5.79
446.15378	1	66085.7	100
447.156	1	18502.9	28
448.15603	1	5832.3	8.83

Figure S12. HRMS of compound **6c.**

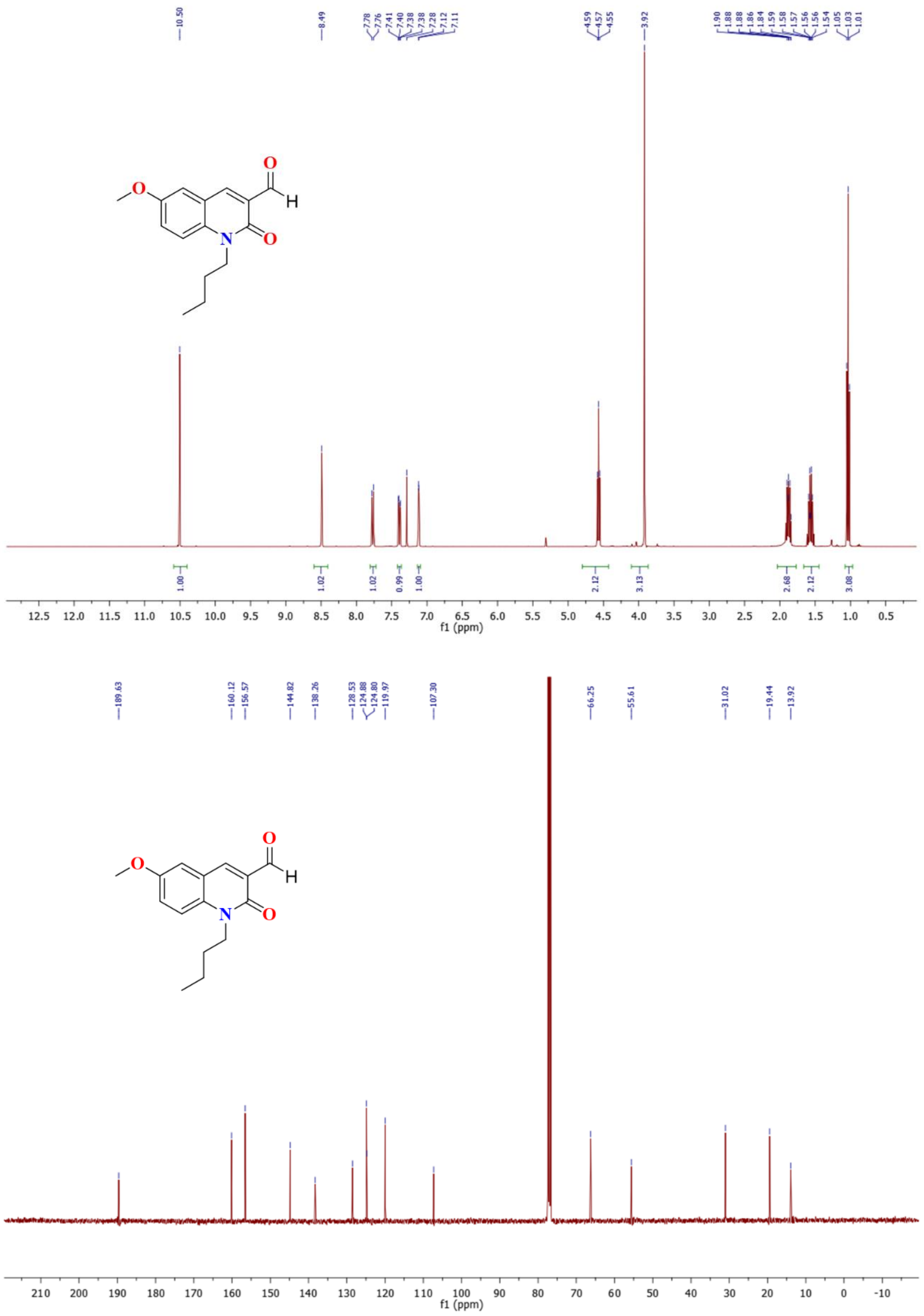

Figure S13. ^{1}H-NMR and ^{13}C-NMR Spectra of compound **4a.**

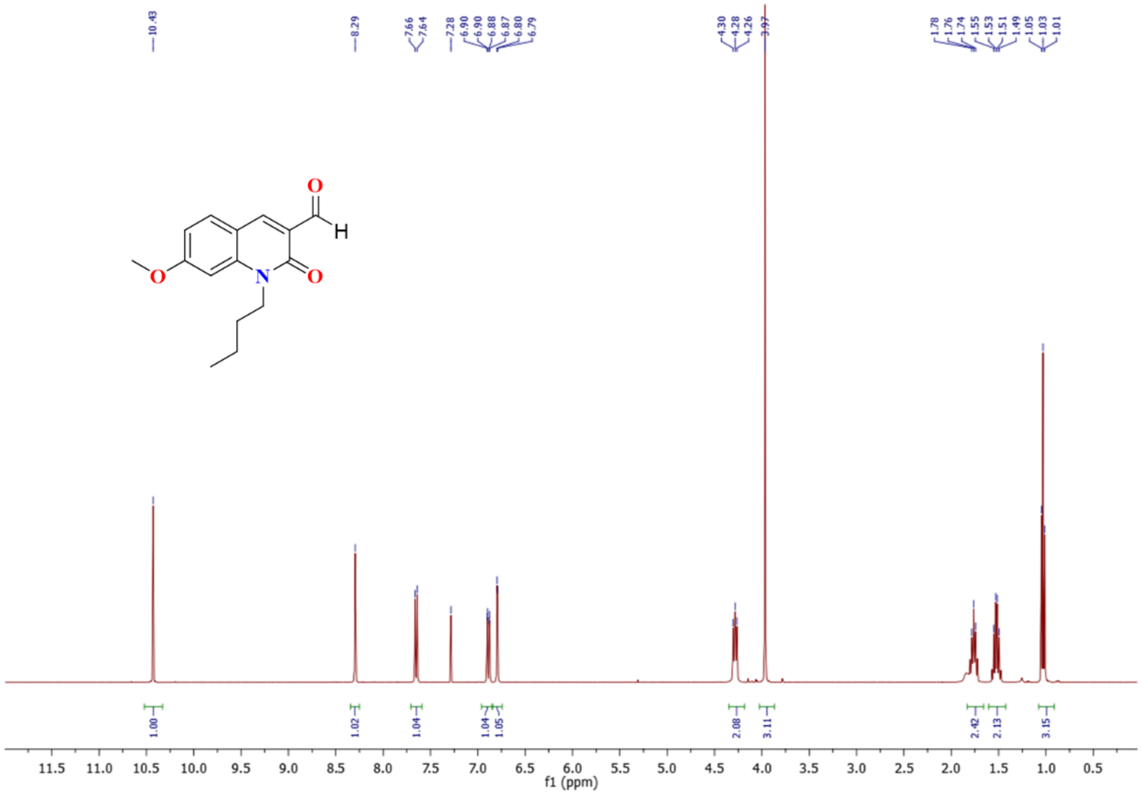

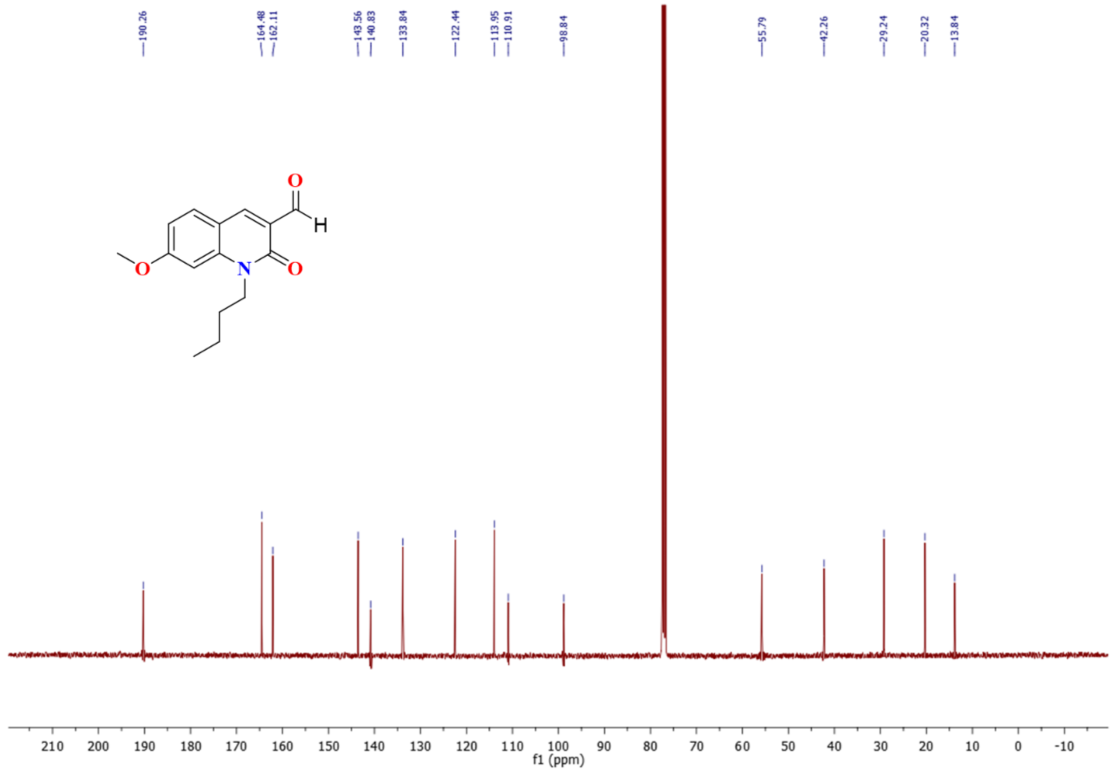

Figure S14. ^{1}H-NMR and ^{13}C-NMR Spectra of compound **4b.**

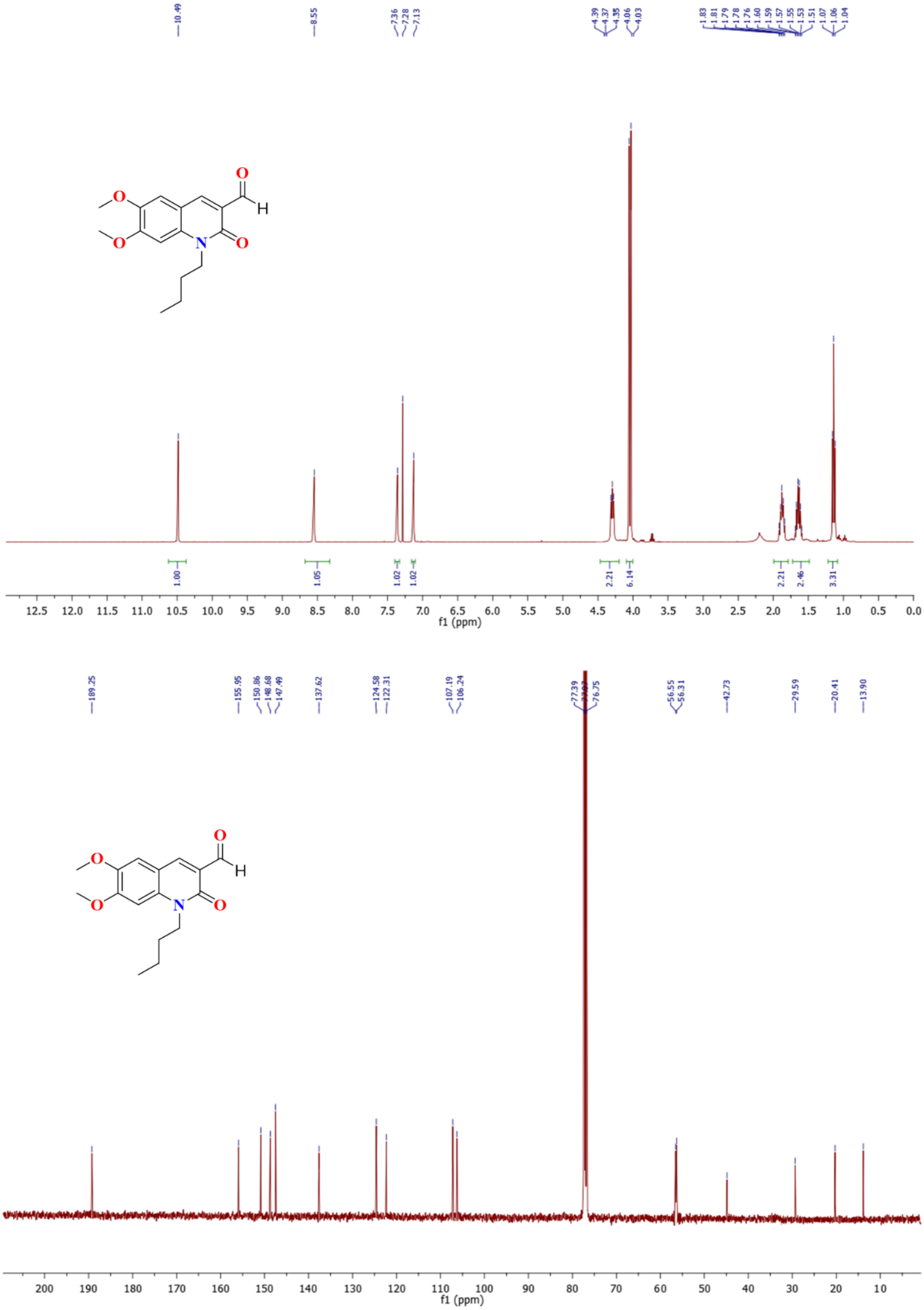

Figure S15. ¹H-NMR and ¹³C-NMR Spectra of compound **4c.**

Table S1. Crystal data and structure refinement for **6c** compound.

**Identification Code**	
Empirical formula	$C_{25}H_{23}N_3O_3S$
Formula weight	445.52
Temperature/K	298
Crystal system	Monoclinic
Space group	$P2_1/c$
a/Å	9.8965(4)
b/Å	17.8620(9)
c/Å	12.6989(5)
α (°)	90
β (°)	96.575(4)
γ (°)	90
Volume/ $Å^3$	2230.04(17)
Z	4
ρ_{calc} mg/mm3	1.327
μ/mm^{-1}	1.554
F(000)	936
2θ range for data collection/°	4.3 to 76.2
Reflections collected	11605
Independent reflections	4620
Data / restraints / parameters	4620/0/292
Goodness-of-fit on F^2	1.05
Completeness (%)	99
Final R indices [I>2sigma(I)]	R1 = 0.0531, wR2 = 0.1652
Largest diff. Peak/hole/ e.$Å^{-3}$	-0.32 and 0.33
